# Optical Polymorphism of Liquid–Crystalline Dispersions of DNA at High Concentrations of Crowding Polymer

**DOI:** 10.3390/ijms241411365

**Published:** 2023-07-12

**Authors:** Vladimir N. Morozov, Mikhail A. Klimovich, Anna V. Shibaeva, Olga N. Klimovich, Ekaterina D. Koshevaya, Maria A. Kolyvanova, Vladimir A. Kuzmin

**Affiliations:** 1 Emanuel Institute of Biochemical Physics, Russian Academy of Sciences, 4 Kosygina, 119334 Moscow, Russia; 2 Burnazyan Federal Medical Biophysical Center, Federal Medical Biological Agency of the Russian Federation, 23 Marshala Novikova, 123182 Moscow, Russia

**Keywords:** molecular crowding, liquid–crystalline DNA, condensed DNA, B–Z transition, molecular hydration, SYBR Green I, Hoechst 33258, circular dichroism

## Abstract

Optically active liquid–crystalline dispersions (LCD) of nucleic acids, obtained by polymer- and salt-induced (*psi*-) condensation, e.g., by mixing of aqueous saline solutions of low molecular weight DNA (≤10^6^ Da) and polyethylene glycol (PEG), possess an outstanding circular dichroism (CD) signal (so-called *psi*-CD) and are of interest for sensor applications. Typically, such CD signals are observed in PEG content from ≈12.5% to ≈22%. However, in the literature, there are very conflicting data on the existence of *psi*-CD in DNA LCDs at a higher content of crowding polymer up to 30–40%. In the present work, we demonstrate that, in the range of PEG content in the system above ≈24%, optically polymorphic LCDs can be formed, characterized by both negative and positive *psi*-CD signals, as well as by ones rather slightly differing from the spectrum of isotropic DNA solution. Such a change in the CD signal is determined by the concentration of the stock solution of PEG used for the preparation of LCDs. We assume that various saturation of polymer chains with water molecules may affect the amount of active water, which in turn leads to a change in the hydration of DNA molecules and their transition from B-form to Z-form.

## 1. Introduction

It is well known that DNA (as well as RNA) molecules in solution may undergo a transition from isotropic to condensed state in the presence of some critical concentrations of cationic salt and crowding polymer. The corresponding technique called *psi*- or ψ-condensation [1,2] is based on the neutralization of negative charges of phosphate groups by counterions, which reduces the DNA–DNA electrostatic repulsion, and the exclusion of nucleic acid phase by DNA-immiscible polymer. For this, a variety of simple salts (e.g., NaCl, KCl, RbCl, etc.) may be used [3,4,5], while the choice of crowding agent is quite limited. The cosolute must satisfy several requirements [6]: (1) solubility in water and aqueous saline solutions in a wide range of concentrations; (2) absence of optical activity and chromophore groups that overlap the absorption region of nitrogenous bases; (3) inertness with respect to nucleotides (lack of precipitation and strong binding). So, nowadays, the most used crowding polymer for this task is polyethylene glycol (PEG).

**Scheme 1:**
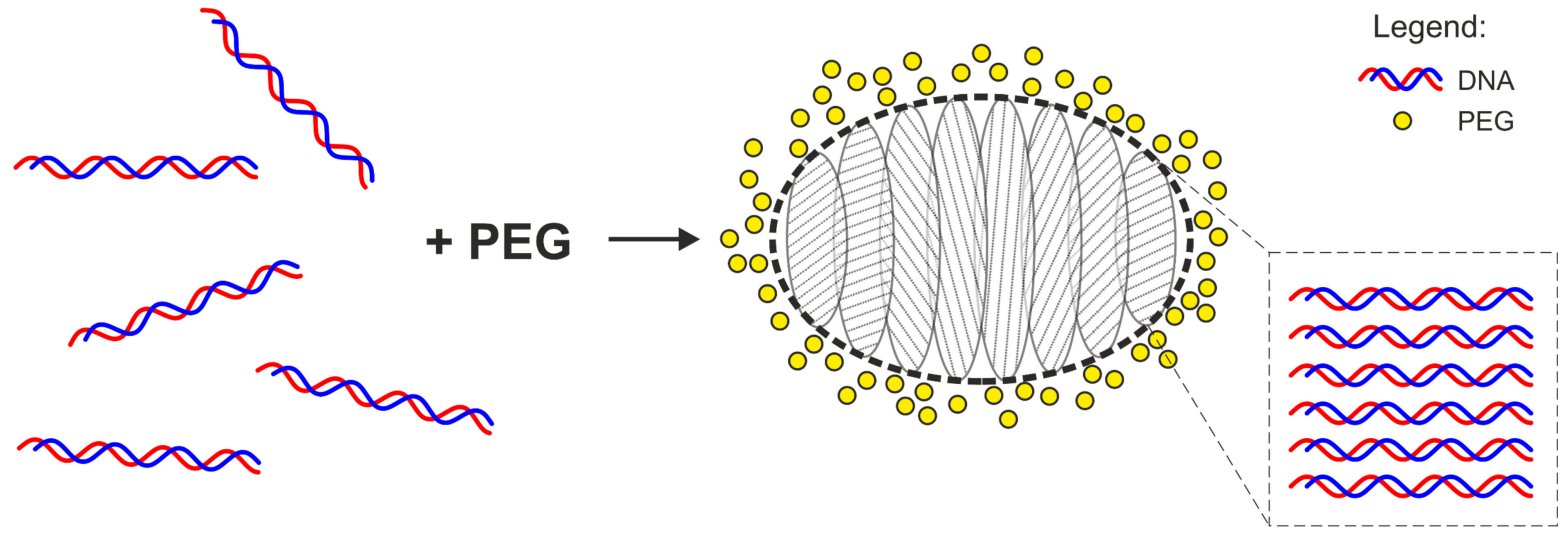
Schematic representation of the formation of DNA CLCD particle as a result of the phase exclusion of DNA molecules from aqueous saline solution upon the addition of PEG (molecules of salt are not shown).

The *psi*-condensation method is universal and can be used both for crowding of single DNA molecules of high molecular weight (monomolecular collapse) [7,8,9,10] and for groups of low molecular weight DNA (multimolecular collapse) [11,12,13]. In the latter case, DNA molecules may form micro- and submicroparticles with a spatially ordered internal structure (experimentally established that it corresponds to cholesteric or hexagonal liquid-crystalline state), which can be controlled by varying the preparation conditions [5]. In the literature, such systems are referred to as liquid–crystalline dispersions (LCDs) of DNA. Under certain conditions, DNA LCDs may have an outstanding optical activity, which is expressed, among other things, in characteristic “anomalies” in the circular dichroism (CD) spectrum (also called ψ type CD or *psi*-CD): a multiply amplified signal inside the DNA absorption range (usually at λ = 260–290 nm) and a long “tail” outside the absorption region [14]. The phenomenon of the “anomalous” optical activity is associated to the specific organization of individual dispersion particles: inside them, DNA molecules constitute parallel layers, rotated relative to each other at a certain angle and, thus, forming a spiral twist typical for cholesteric mesophases (Scheme 1). Dispersions of this type, called cholesteric LCDs (CLCDs), are of interest for optical sensor applications. For example, in recent works, we have shown the possibility of using *psi*-CD as a precise marker of radiation exposure in a wide range of doses [15,16].

At the same time, *psi*-CD is very sensitive to the conditions of DNA CLCD preparation–significant variations in magnitude, shape, sign of the band, as well as the peak position may take place when changing the ratio of the key components of the system or when changing their parameters [5]. A high-intensity CD signal is typically observed for CLCDs formed with DNA molecules of ≈3 × 10^4^ to ≈3 × 10^6^ Da; however, it depends intricately on their structure. The formation of DNA CLCDs also depends in a complex way on the nature of cations: for example, ceteris paribus; the CD signals of the dispersions obtained in the presence of NaCl and MgCl_2_ have opposite orientation. In addition, the internal organization of dispersion particles is determined by the osmotic properties of the medium. Yevdokimov et al. showed that at fixed concentrations of DNA and salt, a change in PEG (M*_w_* = 4 kDa) content leads to a sequential transition of the system from isotropic state to cholesteric mesophase at PEG content of ≈12.5%, and then to the hexagonal one at PEG content of ≈22%. The *psi-*CD in this case exists only in the cholesteric system (12.5–22% of PEG; the “classic” case is the negative band with maximum at λ = 260–280 nm, multiply exceeding by the amplitude the CD signal of isotropic DNA) and is absent in the hexagonal (≥22% of PEG) [17]. The absence of *psi*-CD signal at PEG concentration above 22% was also noticed in [18,19]. At the same time, Goldar et al. noted an increase in the step of cholesteric twist as the concentration of PEG (M*_w_* = 6 kDa) increased from 5% to 15% [20]. The same pitch-unwinding effect with increasing osmotic pressure was found in [21,22].

Nevertheless, the CD signals that are uncharacteristic neither for a dilute solution of B-form DNA nor for the “classic” cholesteric system were observed at high concentrations of PEG in several works. For example, the presence of a small positive band in the wavelength range of 270–290 nm was noted in works by Yevdokimov et al. [23,24]. As an explanation, the authors suggested the appearance of fragments corresponding to the A-form DNA within the LCD particles [24]. Moreover, intense negative and positive CD signals depending on the DNA nucleotide sequence were found in the presence of 40% of PEG (M*_w_* = 1.5 kDa) in work by Sakurai et al. [25]. Thus, an issue of the existence of *psi*-CD signal for molecularly organized DNA ensembles at high concentrations of PEG remains insufficiently studied, and the origin of the observed signals is not entirely clear. Therefore, the purpose of the present work is to investigate the optical activity of DNA LCDs formed in the presence of PEG at concentrations outside the generally accepted region for the formation of cholesteric DNA microparticles and to evaluate its relationship with their internal organization.

## 2. Results and Discussion

**Figure 1:**
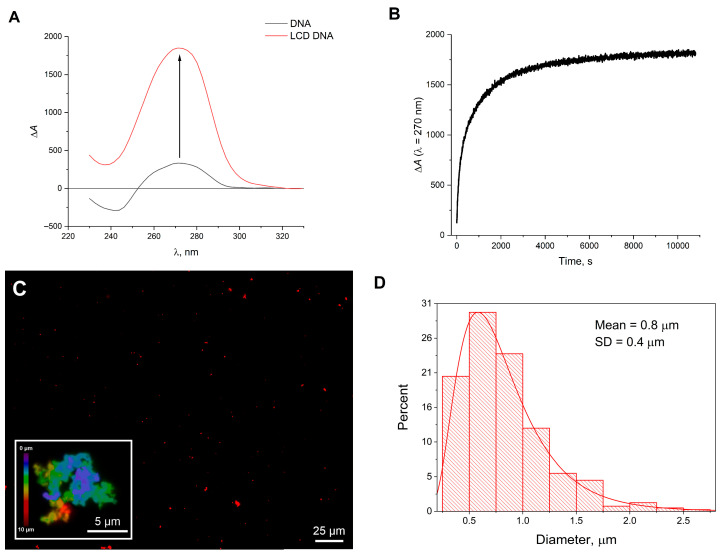
(**A**) CD spectra of isotropic DNA solution (black) and DNA LCD obtained by mixing 1:1 *v*/*v* aqueous saline solutions of DNA and 60% PEG (red). (**B**) The corresponding kinetics of DNA LCD formation: evolution of the CD signal at λ = 270 nm after injection of PEG in the DNA solution. (**C**) Typical microphotograph of the DNA LCD particles stained with 1.44 × 10^−6^ M of SYBR Green I (SG). (**Inset**) Three-dimensional representation of a typical particle of type II. (**D**) Size distribution of the dispersion particles of type I obtained by analysis of the microphotographs.

As noted above, DNA LCDs usually do not have characteristic *psi*-CD signal in the range of PEG concentration of more than ≈22% [17]. Therefore, we were surprised to find an intense positive band in the CD spectrum of the system containing 30% of PEG. The band has a maximum at λ ≈ 270 nm and is ≈5.5-fold higher than the positive signal of isotropic DNA registered in the absence of PEG (Figure 1A). The corresponding CD kinetics recorded after mixing 1:1 *v*/*v* aqueous saline solutions of DNA and 60% PEG (Figure 1B) has a double exponential character cognate to the “classic” DNA CLCD [13]. The values of *k*_1_ and *k*_2_ are 4.0 × 10^−3^ and 4.5 × 10^−4^ s^−1^, respectively. They are noticeably lower than those found in our recent work, which can be explained by the significantly higher viscosity of the medium due to the increased concentration of PEG (30% vs. 17%). The morphology of the dispersion particles and their size distribution, determined using confocal microscopy (Figure 1C,D), also differ from the case of the “classic” cholesteric system. Two types of particles are observed: type I—small spherical particles with average diameter of 0.8 ± 0.4 µm (for the “classic” DNA CLCD, the average diameter is 1.9 ± 0.6 µm [15]); type II—large aggregates of irregular shape similar to grape bundles (often more than 3–4 microns; Figure 1C, inset). At lower PEG content, the particles of this kind are rarely observed. Nevertheless, this phenomenon is in good agreement with the microscopy data that we obtained earlier for the DNA LCD with 34% content of PEG [11].

**Figure 2:**
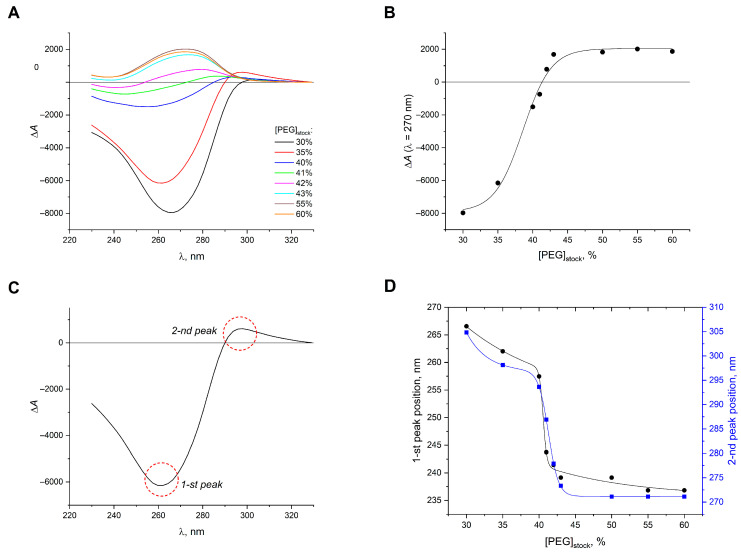
(**A**) CD spectra of DNA LCDs with [PEG]_final_ of 30% prepared using stock solutions of various [PEG]_stock_: 30% (black), 35% (red), 40% (blue), 41% (green), 42% (magenta), 43% (cyan), 55% (brown), 60% (orange). (**B**) Dependence of the CD band intensity at λ = 270 nm on [PEG]_stock_. (**C**) Representation of two characteristic peaks in the CD spectrum on the example of the DNA LCD prepared using [PEG]_stock_ = 35%. (**D**) The position of the peaks as a function of [PEG]_stock_ (first peak—black; second peak—blue).

Although mixing DNA and PEG solutions in 1:1 *v*/*v* ratio is a traditional way for the preparation of DNA LCDs [5], the other ratios of these components may be used. We scrutinized the preparation of DNA LCDs with a final PEG content of 30% from the stock polymer solutions with [PEG]_stock_ concentration from 30% to 60%. The CD spectra of the DNA LCDs obtained in this manner are shown in Figure 2A. It is clearly seen that a change in the stock concentration of PEG has a critical effect on the *psi*-CD signal. For example, CD signals similar to the case of the “classic” cholesteric system are observed for the stock solutions with [PEG]_stock_ ≤ 40%. The corresponding spectra are characterized by intense negative bands exceeding the signal of isotropic DNA from ≈5-fold to ≈28-fold. The CD signal of the dispersion obtained using 30% stock solution is the most intense; increase in the PEG concentration in stock solution to 40% leads to the significant drop in the band amplitude. At [PEG]_stock_ of 41–42%, CD spectra of the obtained DNA LCDs are characterized by two low-intensity differently directed peaks slightly exceeding the signals of isotropic DNA. A further increase in the stock concentration of PEG leads to the appearance of a pronounced positive signal in the CD spectrum. It significantly exceeds the signal of isotropic system, but, at the same time, its amplitude is inferior in absolute value to that of the “classic” DNA CLCD. In the range of [PEG]_stock_ of 43–60%, no significant differences in the CD signals of the DNA LCDs are observed.

The dependence of the intensity of the CD signal at λ = 270 nm on [PEG]_stock_ is well described by a step function and is characterized by a narrow region of transition from negative to positive band orientation (Figure 2B). Thus, the change in the band amplitude from –2000 to +2000 corresponds to the change in [PEG]_stock_ from ≈40% to ≈43%.

The observed differences manifest themselves not only in the variation of amplitude and orientation of the CD signal, but generally in the spectrum shape. In the example of the spectrum of DNA LCD sample prepared using 35% PEG stock solution (Figure 2C), two pronounced peaks can be noted: first—an intense negative one with maximum at λ_1_ = 262 nm; second—a little positive one with maximum at λ_2_ = 298 nm. We assume that in the studied system the intense positive peak at λ ≈ 270 nm, observed at high stock concentrations of PEG (43–60%), is not identical to the first peak taken with the opposite sign and differed by the amplitude, but stems precisely from the second peak. The peaks synchronously shift towards short wavelengths with an increase in [PEG]_stock_: the first one shifts from 267 to 237 nm, and the second from 305 to 271 nm, respectively (Figure 2D). Herewith, the intensity of the first peak drops rapidly, while the intensity of the second eventually increases (the ratio of the absolute values of their amplitudes changes from ≈57 at [PEG]_stock_ = 30% to ≈0.15 at [PEG]_stock_ = 60%).

Typically, the “tail” in the *psi*-CD spectrum, which appears due to light scattering, lies in the region above 300 nm [14]; therefore, the positive peak observed in the range of λ = 271–305 nm may be attributed to the structural features of the DNA LCD particles. The similar particle sizes of the dispersions obtained using 30% and 60% stock solutions of PEG strongly indicates in favor of this assumption. At the same time, the literature data provide a very mixed picture on this issue. Yevdokimov et al., who studied DNA LCDs in detail, also observed a relatively small positive CD signal close to λ ≈ 290–300 nm along with an intense negative peak at λ ≈ 260–270 nm [26,27]. For example, a similar effect with the appearance of such CD signal was observed during the treatment of the “classic” DNA CLCD with platinum compounds [28]. However, there was no such CD signal in many other works of this group (e.g., in [29]).

**Scheme 2:**
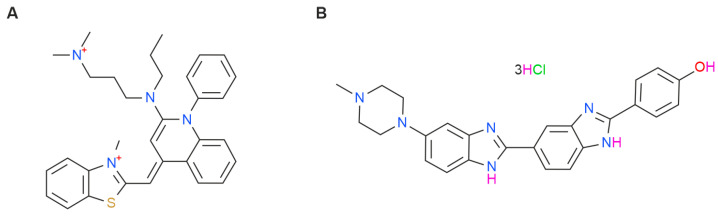
Structures of SG (**A**) and Ht58 (**B**).

**Figure 3:**
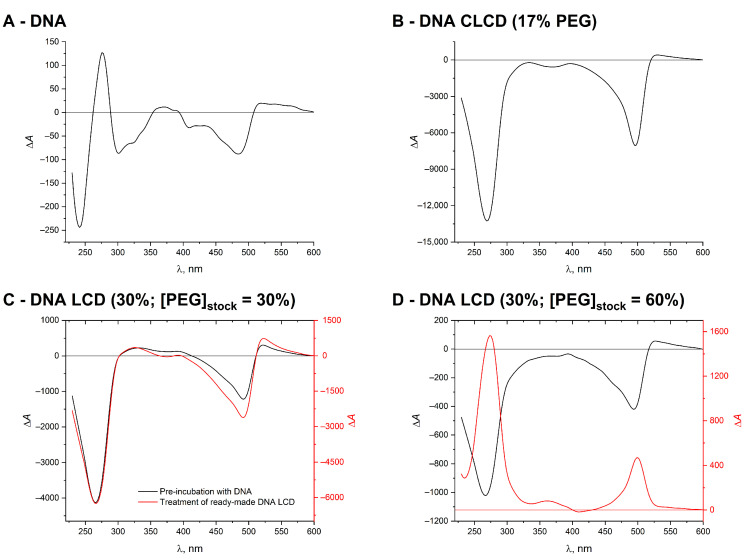
CD spectra of isotropic DNA solution (**A**), “classic” DNA CLCD with 17% PEG content (**B**), and DNA LCDs with 30% PEG content, prepared using stock solutions of [PEG]_stock_ = 30% (**C**) or [PEG]_stock_ = 60% (**D**), respectively. All systems were treated with 7 × 10^−6^ M of SG. (**C**,**D**) show the comparison of the preincubation of the dye with DNA followed by the addition of PEG and the formation of the dispersion systems (black) and its introduction into the ready-made DNA LCDs (red).

**Figure 4:**
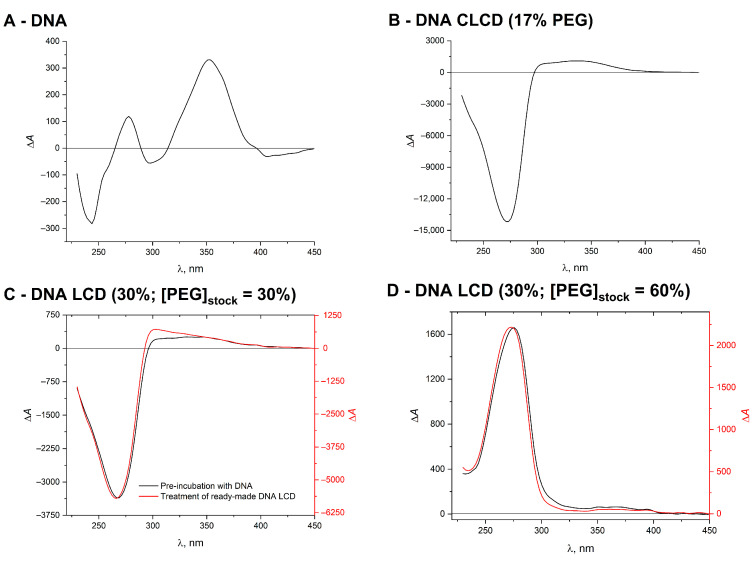
CD spectra of isotropic DNA solution (**A**), “classic” DNA CLCD with 17% PEG content (**B**), and DNA LCDs with 30% PEG content, prepared using stock solutions of [PEG]_stock_ = 30% (**C**) or [PEG]_stock_ = 60% (**D**), respectively. All systems were treated with 7 × 10^−6^ M of Ht58. (**C**,**D**) show the comparison of the preincubation of the dye with DNA followed by the addition of PEG and the formation of the dispersion systems (black) and its introduction into the ready-made DNA LCDs (red).

Figure 3 and Figure 4 show a comparison of the CD spectra of isotropic DNA solution, “classic” DNA CLCD with 17% PEG content, and DNA LCDs with 30% PEG content, prepared using the polymer stock solutions of 30% or 60%, stained with 7 × 10^−6^ M of SG or Hoechst 33258 (Ht58) in each case. These dyes are well-known DNA-specific ligands (their structures are shown in Scheme 2): SG binds to double-stranded DNA predominantly in an intercalation mode [30], while Ht58 molecules are localized in the minor groove of the double helix [31]. Both dyes are optically inactive in free state, but their binding to a chiral biomolecular matrix cause the appearance of the induced circular dichroism (ICD). The complex of SG with DNA is characterized by a small negative ICD peak with minimum at λ ≈ 500 nm (Figure 3A), while the complex formation between Ht58 and DNA is accompanied by the appearance of an intense positive ICD band with maximum at λ ≈ 350 nm (Figure 4A). In the “classic” DNA CLCD, on the contrary, the DNA–Ht58 complex corresponds to a significantly less intense ICD band than that of SG (Figure 3B and Figure 4B). In this case, the ICD signal of SG is codirected with the main *psi*-CD band at λ ≈ 270 nm, while the signal of Ht58 is directed on the opposite side. Note that, in these cases, the dyes were added to the ready-made dispersion systems.

For the DNA LCDs with 30% final content of PEG, two different methods of dye addition were tested: treatment of the ready-made liquid–crystalline systems or preincubation of SG and Ht58 with DNA prior to the injection of PEG and formation of the dispersion particles. Regardless of the order of dye addition, the CD spectra of DNA LCDs prepared using 30% stock solution of PEG, and stained with SG, are similar in shape to that of the “classic” DNA CLCD (Figure 3C). In both cases, the addition of the dye results in a decrease in the amplitude of the main *psi*-CD band at λ ≈ 270 nm. The greatest difference in the native signal (about 2-fold) is observed for the case of preliminary incubation of SG with DNA (vs. ≈1.3-fold decrease in the main *psi*-CD band intensity in the case of staining of the ready-made DNA LCD). In the case of the DNA LCDs prepared using 60% stock solution of PEG, the positively oriented CD signals are observed only for the ready-made system treatment. Otherwise, binding of SG to DNA prior to the crowding interferes with the formation of the dispersion system with positive CD signals (Figure 3D): only low-amplitude negative signals are observed in the CD spectrum in this case. In turn, addition of Ht58 leads to a pronounced ICD signal only in the DNA LCD prepared using 30% stock solution of PEG and only in the case of the dye injection into the ready-made dispersion system (Figure 4C). In the case of preincubation of the dye with DNA, as well as in case of the DNA LCD preparation using 60% stock solution of PEG (regardless of the order of the dye addition; Figure 4D), no pronounced ICD signals are detected. Similar to SG, addition of Ht58 results in the decrease in the main *psi*-CD band amplitude for the case of DNA LCDs prepared using 30% stock solution of PEG.

**Figure 5:**
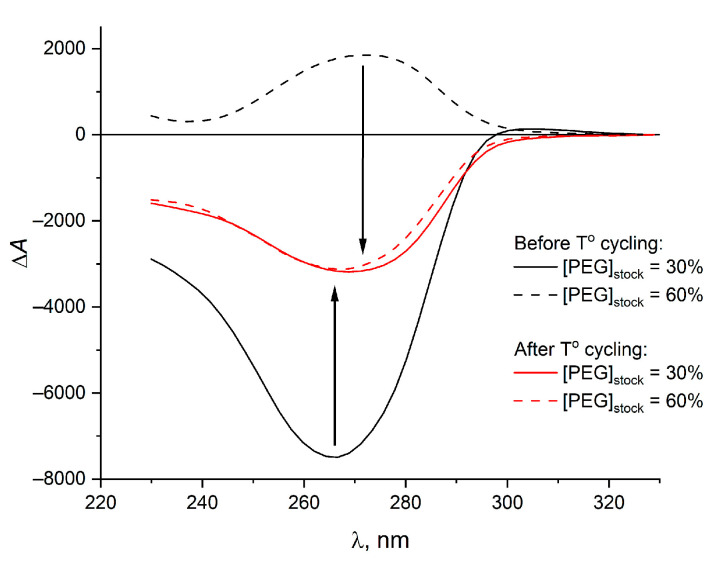
CD spectra of DNA LCDs with 30% PEG content, prepared using stock solutions with polymer concentration of 30% (solid) and 60% (dashed), before (black) and after (red) the heat treatment of the samples.

Further, the so-called thermal “hardening” was performed for DNA LCDs prepared using stock solutions with [PEG]_stock_ of 30% or 60%. This involves heating of the samples to 80 °C and their subsequent cooling to 20 °C (in the case of the “classic” DNA CLCD, such T° cycling usually leads to a noticeable increase in the CD signal) [32]. The corresponding CD spectra of the studied systems before and after the heat treatment are shown in Figure 5. Before the treatment, their CD signals were directed on opposite sides: the larger negative peak matches the DNA LCD prepared using 30% stock solution of PEG, and the lower amplitude positive band matches that prepared using 60% polymer stock solution. In both cases, the CD signals decrease with increasing the temperature until a complete disappearance of the anomalous bands at 80 °C. Cooling of the samples, in turn, leads to the appearance in both systems of similar negative CD peaks in the region of 266–271 nm. In absolute values, the amplitude of the resulting signals is ≈2.4-fold less than the amplitude of the initial negative band, and ≈1.7-fold greater than that of the initial positive band. Note that there are no positive signals in the region of 290–300 nm after the samples cooling.

**Figure 6:**
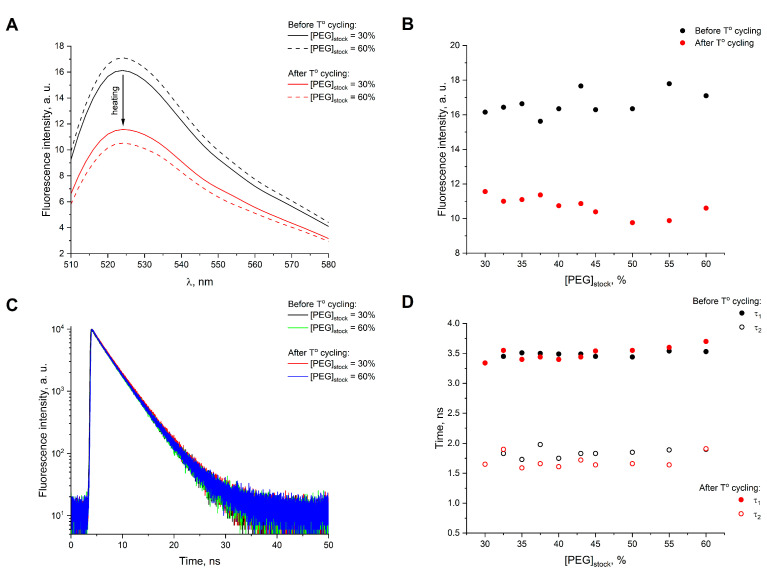
(**A**) Fluorescence spectra of 3.50 × 10^−6^ M SG in DNA LCDs with 30% PEG content, prepared using stock solutions of [PEG]_stock_ = 30% (solid) or [PEG]_stock_ = 60% (dashed), before (black) and after the heat treatment (red). (**B**) Dependence of the maximum SG fluorescence intensity on [PEG]_stock_ for DNA LCD samples before (black) and after (red) the heat treatment. (**C**) Decay kinetics of 3.50 × 10^−6^ M SG fluorescence (λ_fl_ = 520 nm): black and green—[PEG]_stock_ = 30% and [PEG]_stock_ = 60% before T° cycling; red and blue—the same stock concentration of PEG after T° cycling. (**D**) Dependence of τ_1_ (filled) and τ_2_ (hollow) values on [PEG]_stock_ for DNA LCD samples before (black) and after (red) the heat treatment.

The change in the CD signal observed when varying the stock concentration of PEG may indicate a change in the packaging density of DNA molecules in the particles of resulting dispersions. It is well known that DNA molecules in solution can exist in a variety of phases and undergo transitions between them: isotropic ↔ blue phases (or precholesteric stages) ↔ cholesteric phase ↔ 2D and 3D hexagonal phases ↔ orthorhombic phase [33,34], wherein the distances between adjacent DNA molecules in liquid–crystalline phases vary from ≈4.9 to ≈2.4 nm, and the cholesteric phase usually exists at the distances of 4.9–3.2 nm [35]. Thus, the decrease in the CD signal amplitude may be associated with an expansion of the intermolecular space or with a compaction of the DNA molecules. At the same time, a change in the packaging density of DNA molecules may contribute to a change in a fluorescence intensity of bound dye [36]. Based on these two facts, using SG as a fluorescent probe, we assessed the change in DNA packaging under different conditions of LCDs preparation. It was assumed that an increase in the packaging density of DNA molecules would be accompanied by a quenching of SG fluorescence, while an increase in the distances between neighboring DNA molecules would be accompanied by an increase in emission intensity of the dye. Fluorescence spectra of 3.50 × 10^−6^ M SG, as well as corresponding decay curves, were recorded before and after the temperature treatment of the DNA LCD samples for all studied values of [PEG]_stock_ (in all cases, the dye was added to the ready-made DNA LCD). The results are shown in Figure 6. It is clearly seen that the SG fluorescence intensity remains unchanged when using various stock concentrations of PEG for DNA LCD preparation. The fluorescence intensity decreases by ≈35% only after T° cycling, but in this case, it also does not depend on [PEG]_stock_. Together with foregoing data on the thermal “hardening” of DNA LCDs, this allows to conclude that, in the studied case, the packaging density of DNA molecules in dispersion particles become higher after heat treatment. At the same time, the lifetime of the SG’s excited state does not change neither with varying [PEG]_stock_ nor after heat treatment. The decay kinetics are two-component. The average values of τ_1_ and τ_2_ are 3.5 ± 0.1 and 1.8 ± 0.1 ns, and their contributions to the total amplitude of SG fluorescence are 60–75% and 25–39%, respectively.

**Figure 7:**
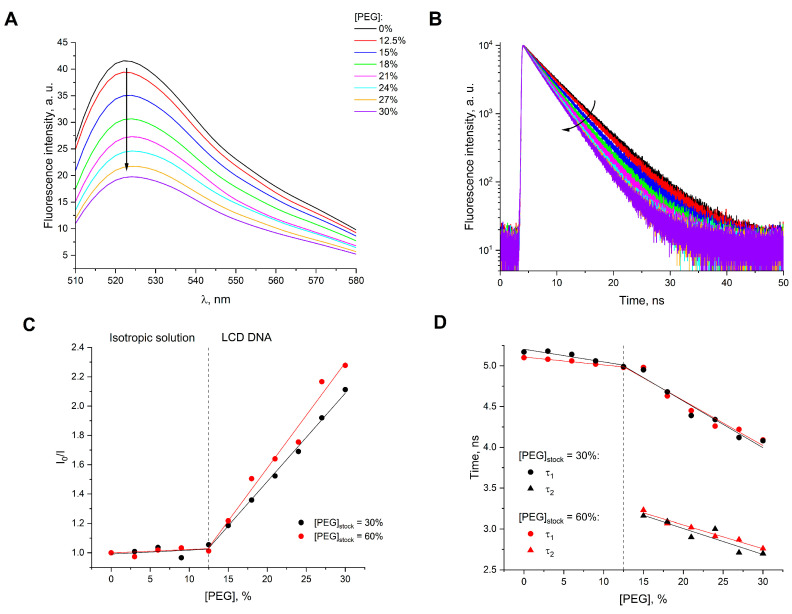
(**A**,**B**) Fluorescence spectra (**A**) and decay kinetics (**B**) of 2.33 × 10^−6^ M SG in the DNA-PEG mixtures with various final content of the polymer: 0% (black), 12.5% (red), 15% (blue), 18% green, 21% (magenta), 24% (cyan), 27% (yellow), 30% (violet). The stock solution of [PEG]_stock_ = 60% was used. (**C**,**D**) The SG fluorescence intensity on [PEG]_final_ in Stern–Volmer coordinates for two different stock solutions of the polymer (**C**) and the corresponding lifetimes (**D**): [PEG]_stock_ = 30% (black) and [PEG]_stock_ = 60% (red). In (**D**) τ_1_ and τ_2_ values are indicated by circles and triangles, respectively.

The results obtained strongly indicate that there is no change in the distances between adjacent DNA molecules/layers in LCD particles prepared using stock solutions of various PEG concentrations. When this parameter changes, it can be clearly seen from the response of the dye fluorescence. As an example, Figure 7 shows the results of investigation of the fluorescent properties of SG in the DNA-PEG mixtures with final polymer concentration from 0% to 30% (the stock solution of [PEG]_stock_ = 60% was used). In all cases, the dye was added to the ready-made system. No significant changes in SG fluorescence are observed until the critical PEG concentration of 12.5% (this value corresponds to the beginning of the LCD formation). Further increase in PEG concentration leads to a gradual decrease in SG fluorescence (Figure 7A). At 30% content of PEG, the fluorescence intensity decreases by more than twofold compared to the control (a higher fluorescence at lower SG concentration compared to Figure 6 seems to be associated with the reduced self-quenching effect [37]). This drop is accompanied by a decrease in the lifetime of excited state of the dye. The corresponding decay curves are shown in Figure 7B. Starting with PEG concentration of 15%, the lifetime becomes two-component. In the range of 15–30% of PEG, τ_1_ and τ_2_ values vary from 3.1 ± 0.1 to 2.7 ± 0.1 ns and from 5.0 ± 0.1 to 4.1 ± 0.1 ns, respectively. The contribution of the short-lived component to the total amplitude of fluorescence signal in this case increases from ≈26% to ≈46%.

Dependences of the SG fluorescence intensity on PEG concentration in Stern–Volmer coordinates, as well as the corresponding dependences of the lifetimes are almost the same within experimental error for two different stock solutions of the polymer (30% and 60%; Figure 7C,D). In general, there are no significant differences between these two cases. Thus, the change in the CD signal is not associated with a change in the packaging of DNA within the LCD particles.

**Figure 8:**
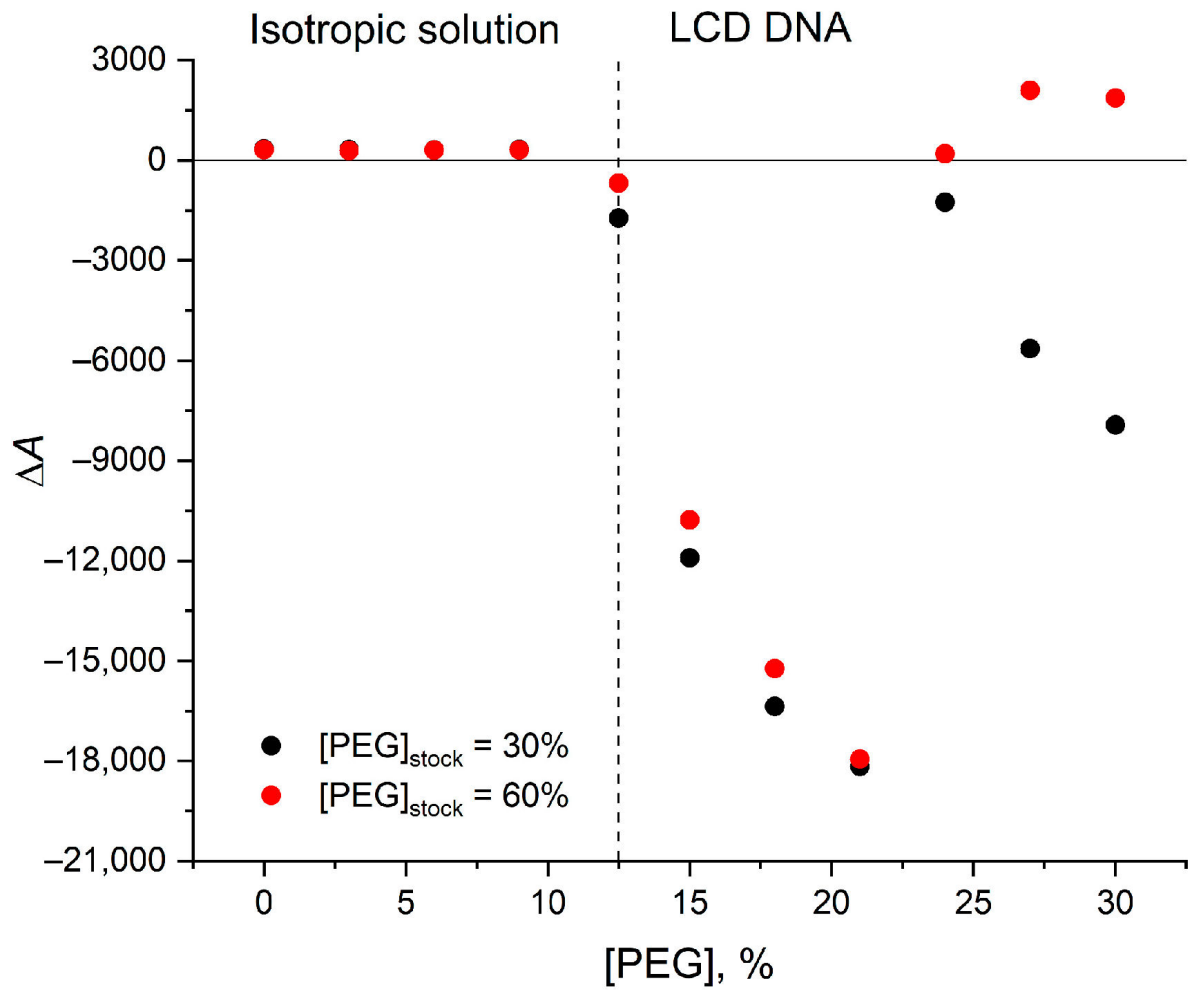
Dependence of the CD band amplitude on the final concentration of PEG in the DNA-PEG mixtures, prepared using its stock solutions with [PEG]_stock_ values of 30% (black) and 60% (red).

Further, we determined the region of existence of the dependence of CD signal on [PEG]_stock_. The amplitudes of CD bands for the DNA-PEG mixtures with final PEG concentration from 0% to 30%, prepared using its stock solutions with concentrations of 30% or 60%, were measured (Figure 8). It is clearly seen that in both cases, the optically active dispersion is not formed at PEG content below 12.5%. In this area and beyond up to ≈24%, the CD signal is almost independent on [PEG]_stock_. However, a further increase in the PEG content leads to noticeable differences. In case of [PEG]_stock_ = 30%, the curve has a kink at 24% content of the polymer and rushes down, while a plateau is observed in case of [PEG]_stock_ = 60%. According to the previously published data, the CD intensity in this region should be close to zero [17].

Thus, based on the results obtained, we conclude that the observed effect of the CD signal inversion is not associated with a change in the osmotic conditions, but may be due to a change in the structure of individual DNA molecules within the LCD particles. We cannot be sure exactly what kind of change in the DNA conformation takes place, so our future work will be devoted to a detailed study of this issue. Nevertheless, we assume that a transition from right-handed B-form to left-handed Z-form may be a reason for this phenomenon. Below are some arguments in support of this hypothesis.

It is well known that the structure of a DNA molecule is influenced by hydration to a great extent. Thus, any changes in the osmotic environment that affect the number of bound and unbound water molecules, at the same time, influence the DNA molecule’s stability and conformation [38,39]. Therefore, as the presence of high PEG concentration in the system significantly lowers water activity, its impact on DNA is associated with both steric crowding and dehydration [40]. Specifically, a decrease in the activity of water has been suggested to be a source of duplex destabilization by increasing PEG concentration in solution [41,42]. At the same time, the B–Z transition also seems to be associated with dehydration of DNA molecules [43,44,45,46]. Thus, since the inversion of *psi*-CD occurs at [PEG]_stock_ > 41%, it can be assumed that at lower concentrations, the polymer chains in its stock solution are more saturated with water molecules, and the solubilization of DNA molecules does not change critically during the preparation of LCDs. On the other hand, at high values of [PEG]_stock_, the polymer chains in the stock solutions are far from saturation with water molecules and, when mixed with DNA solution, they may significantly reduce water activity.

In dilute solutions, the B–Z transition usually occurs at high cation concentrations. However, under the conditions of macromolecular crowding (e.g., in the presence of PEG), it may be much less energy-consuming and require a much lower ion concentration [47,48]. Moreover, Yevdokimov et al. have previously shown that LCD obtained by condensation of Z-form DNA is characterized by a positive *psi*-CD [49]. This effect was observed at NaCl concentration of 3.2 M and at 18% final content of PEG. Considering that an increase in PEG concentration may lead to a decrease in the amount of salt required for the B–Z transition [48], we can assume the possibility of its realization under the studied experimental conditions: NaCl concentration of 0.3 M and 30% final content of PEG. The B–Z transition can be implemented even at much lower salt concentration—using the example of self-assembled branched-DNA nanostructures, Bhanjadeo et al. showed its existence starting with 5 mM of LaCl_3_ [50].

The behavior of DNA-specific dyes may also evidence that the observed phenomenon is associated with a change in the structure of DNA. Thus, preincubation of SG with DNA hinders the formation of LCD with a positive CD signal. It is well known that intercalating dyes not only interfere with DNA condensation [51,52,53], but also inhibit B–Z transition [54,55]. Both effects are mainly due to the structural impact of intercalating dyes on the DNA molecule. It should be noted here that SG has not been studied experimentally in this capacity; however, Hur et al. showed that this dye has much lower fluorescence in complex with Z-form DNA compared to B-form [56]. In the present study, we did not observe such an effect, which can be explained, among other things, by another dye/base pair ratio (0.06 vs. 0.38). Groove binders, in contrast, do not hinder DNA condensation [57]; however, binding of Ht58 to DNA requires unbound water molecules, and the presence of osmolytes significantly reduces the efficacy of complex formation [58,59]. This may explain why we do not observe any clear ICD signals for this dye in the LCDs prepared using 60% stock solutions of PEG.

Finally, the assumption of a change in the conformation of DNA molecules is in good agreement with the principle of alternating-sign hierarchies of chiral structures [60]. In accordance with this principle, right-handed DNA molecules (A- and B-forms) should assemble into a left-handed superstructure characterized by a negative CD signal, while left-handed DNA molecules (Z-form) should form a right-handed system with a positively oriented CD signal, respectively. Since the confocal microscopy data generally demonstrate the inalterability of the LCD particles’ morphology and the results of fluorescence experiments indicate a permanence of the distances between adjacent DNA molecules/layers within them, we conclude that the internal ordered layered structure of LCD particles is preserved when the sign of the CD band is reversed, and orientation of the *psi*-CD signal, in turn, is associated exclusively with the mutual orientation of the DNA layers. At the boundary points, when most of DNA molecules are in B-form or Z-form, the layers are oppositely twisted, while at intermediate points, the system is a mixture of particles with different twisting of the layers.

## 3. Materials and Methods

The commercial DNA preparation from salmon sperm depolymerized ultrasonically (Derinat^®^; (0.25–0.5) × 10^6^ Da (≈400–800 bp); Technomedservice, Moscow, Russia) and PEG (4000 Da; Sigma, St. Louis, MO, USA) were used to make DNA LCD samples. The DNA and PEG solutions were prepared on the basis of aqueous saline buffer containing 0.01 M NaH_2_PO_4_ and 0.3 M NaCl (pH ≈ 7.4). The DNA LCD samples of 1.5 mL volume were prepared in Eppendorf tubes by mixing the DNA and PEG solutions in various proportions. The resulting mixtures were vigorously stirred and held at room temperature for 90–120 min. To obtain a final PEG concentration ([PEG]_final_) of 30% in the DNA LCD samples, the following concentrations of its stock solutions ([PEG]_stock_) were used: 30, 35, 40, 41, 42, 43, 45, 50, 55, and 60%. In all cases DNA concentration in nucleobases in the final sample was 1.2 × 10^−4^ M (24 µL of DNA preparation per 1.5 mL of DNA LCD). For example, in the case of [PEG]_stock_ = 30%, a 24 µL drop of DNA was added directly to 1.5 mL of the polymer solution, and the in case of [PEG]_stock_ = 60%, the mixing of the polymer solution with the aqueous saline solution of DNA was performed in 1:1 *v*/*v* (750:750 µL). Thus, the volume part of PEG solution in the DNA LCD samples ranged from 1.0 to 0.5. Since the volume of DNA preparation was less than 0.02% of the sample volume, it was neglected in concentration calculations.

The CD spectra of the DNA LCD samples were recorded using SKD-2 dichrometer manufactured at the laser spectral instrumentation department of the Institute of Spectroscopy, Russian Academy of Sciences [61]. The measurements were carried out at room temperature in quartz cells with an optical path length of 1 cm. The spectra were presented as the dependence of the difference between an absorption of left- and right-hand polarized light $\Delta A=A_{L}-A_{R}$ on wavelength λ. In the experiments with heat treatment of the DNA LCD samples, they were heated and cooled in a thermostatically controlled cell compartment of the dichrometer. Each sample was heated from 20 °C to 80 °C and then cooled to 20 °C. At the temperature checkpoints, they were kept for 10–12 min for uniform heating/cooling. In the kinetic experiments, the CD signal was recorded at λ = 270 nm (signal at λ = 600 nm was taken as a zero point) immediately after manual mixing of DNA and PEG solutions. The dead time was about 5–7 s. Experimental data were processed using global kinetic analysis with Equation (1):

(1) $$A\left(t\right)=\sum_{i=1}^{n}A_{i}e^{{-k}_{i}t}.$$

The error in determination the rate constants did not exceed 10%.

Commercial DNA-specific dyes SYBR Green I (SG; Lumiprobe, Cockeysville, MD, USA) and Hoechst 33258 (Ht58; Paneko, Moscow, Russia) were used to study an induced circular dichroism (ICD). Concentration of the dyes as well as DNA concentration was determined spectrophotometrically on a Shimadzu UV-3101 PC spectrophotometer (Kyoto, Japan) using the molar absorption coefficients: ε_260_ ≈ 6600 M^−1^ cm^−1^ (DNA) [62], ε_498_ ≈ 73,000 M^−1^ cm^−1^ (SG) [63], ε_340_ ≈ 42,000 M^−1^ cm^−1^ (Ht58) [64]. SG was also used as a fluorescent probe. The fluorescence spectra were recorded using RF5301PC spectrofluorimeter (Shimadzu, Kyoto, Japan). The excitation wavelength was λ = 498 nm, and the emission was recorded in the range of 510–600 nm. The fluorescence decay curves of SG in DNA LCD samples were obtained using FluoTime 300 fluorescence lifetime spectrometer (PicoQuant, Berlin, Germany) with picosecond time-correlated single photon counting (TCSPC). A pulsed 485 nm laser light source at 20 MHz pulsed mode was used for dye excitation; emission was registered at 520 nm. The experimental curves were fitted with the exponential model using EasyTau 2 software. The weighted mean-square deviation *χ*^2^ in all cases did not exceed 1.3. In the experiments with heat-treated samples, SG was added after the T° cycling when the samples were completely cooled. All fluorescence measurements were performed in 1.0 × 0.4 cm quartz cells with 1 cm path length of excitation light at room temperature.

Visualization of the DNA LCD particles was performed using Leica TCS SP5 confocal laser scanning microscope with LAS AF software (v. 2.7.3; Leica Microsystem GmbH, Wetzlar, Germany). A 50 µL drop of DNA LCD sample stained with 1.44 × 10^−6^ M of SG was placed on a glass slide and covered with the cover slip. An Ar 488 nm laser was used for excitation the dye; the fluorescence was detected in the wavelength range of 500–600 nm. The analysis of particle size was performed using ImageJ software (v. 1.53; National Institutes of Health, Bethesda, MD, USA). At least 300 particles of the DNA LCD were processed in each case.

## 4. Conclusions

In the present work, we showed, for the first time, that DNA LCDs with pronounced optical polymorphism can be obtained at PEG content above the generally accepted range characteristic for the formation of optically active DNA microparticles (≥24%). These systems either have intense negative CD bands—which are typical for the cholesteric mesophases of B-form DNA and multiply exceed the CD signal of isotropic DNA in amplitude—or are characterized by completely different spectra. For example, CD spectra of the DNA LCD samples may have two low-intensity, differently directed peaks that slightly exceed the signals of isotropic DNA. Moreover, in some cases, the LCDs may have a pronounced positive signal in the CD spectrum. It significantly exceeds the signal of isotropic system, but, at the same time, its amplitude is inferior in absolute value to that of the “classic” DNA CLCD. Such a change in the CD signal is determined by the concentration of the stock solution of PEG used for the preparation of the dispersed systems.

Since the confocal microscopy data generally demonstrate the inalterability of LCD particles’ morphology and the results of fluorescence experiments indicate a permanence of the distances between adjacent DNA molecules/layers within them, we conclude that the observed effect of the CD signal inversion is not associated with a change in the internal ordered layered structure of the LCD particles but is due to a change in the structure of individual DNA molecules. We assume that various saturation of polymer chains with water molecules in stock solutions affects the amount of active water, which in turn leads to a change in the hydration of DNA molecules and causes their transition from B-form to Z-form. At the boundary points, when most of the DNA molecules are in B-form or in Z-form, the layers are oppositely twisted, while at intermediate points, the system is a mixture of particles with different twisting of the layers.

The data obtained may be in demand, since, in our opinion, DNA LCDs are convenient model systems for the investigation the interaction of small ligands and nanomaterials with DNA under conditions close to those existing in some biological systems. The ability to change the conformation of DNA molecules will successfully complement the possibility of varying such parameters in these systems as the local concentration of the nucleic acid, the degree of ordering of the DNA molecules, as well as their packaging density (if, of course, the hypothesis proposed in the present paper will be confirmed in further experiments). In addition, the found phenomenon of the optical polymorphism may be of interest from the point of view of the applications of DNA LCDs in sensorics.

## Data Availability

The data that support the findings of this study are available from the corresponding author upon reasonable request.

## References

[1] Lerman L.S. (1971). A transition to a compact form of DNA in polymer solutions. Proc. Natl. Acad. Sci. USA.

[2] Jordan C.F., Lerman L.S., Venable J.H. (1972). Structure and circular dichroism of DNA in concentrated polymer solutions. Nat. New Biol..

[3] Katsura S., Hirano K., Matsuzawa Y., Yoshikawa K., Mizuno A. (1998). Direct laser trapping of single DNA molecules in the globular state. Nucleic Acids Res..

[4] Zinchenko A.A., Yoshikawa K. (2005). Na+ shows a markedly higher potential than K+ in DNA compaction in a crowded environment. Biophys. J..

[5] Yevdokimov Y.M., Salyanov V.I., Semenov S.V., Skuridin S.G. (2011). DNA Liquid-Crystalline Dispersions and Nanoconstructions.

[6] Nakano S.I., Miyoshi D., Sugimoto N. (2014). Effects of molecular crowding on the structures, interactions, and functions of nucleic acids. Chem. Rev..

[7] Vasilevskaya V.V., Khokhlov A.R., Matsuzawa Y., Yoshikawa K. (1995). Collapse of single DNA molecule in poly(ethylene glycol) solutions. J. Chem. Phys..

[8] Hirano K., Ichikawa M., Ishido T., Ishikawa M., Baba Y., Yoshikawa K. (2012). How environmental solution conditions determine the compaction velocity of single DNA molecules. Nucleic Acids Res..

[9] Ojala H., Ziedaite G., Wallin A.E., Bamford D.H., Hæggström E. (2014). Optical tweezers reveal force plateau and internal friction in PEG-induced DNA condensation. Eur. Biophys. J..

[10] Cheng C., Jia J.L., Ran S.Y. (2015). Polyethylene glycol and divalent salt-induced DNA reentrant condensation revealed by single molecule measurements. Soft Matter..

[11] Morozov V.N., Kolyvanova M.A., Dement’eva O.V., Rudoy V.M., Kuzmin V.A. (2020). Fluorescence superquenching of SYBR Green I in crowded DNA by gold nanoparticles. J. Lumin..

[12] Morozov V.N., Kolyvanova M.A., Dement’eva O.V., Rudoy V.M., Kuzmin V.A. (2021). Comparison of quenching efficacy of SYBR Green I and PicoGreen fluorescence by ultrasmall gold nanoparticles in isotropic and liquid-crystalline DNA systems. J. Mol. Liq..

[13] Morozov V.N., Klimovich M.A., Kostyukov A.A., Belousov A.V., Kolyvanova M.A., Nekipelova T.D., Kuzmin V.A. (2022). Förster resonance energy transfer from Hoechst 33258 to SYBR Green I in cholesteric liquid-crystalline DNA. J. Lumin..

[14] Keller D., Bustamante C. (1986). Theory of the interaction of light with large inhomogeneous molecular aggregates. II. Psi-type circular dichroism. J. Chem. Phys..

[15] Kolyvanova M.A., Klimovich M.A., Shibaeva A.V., Koshevaya E.D., Bushmanov Y.A., Belousov A.V., Kuzmin V.A., Morozov V.N. (2022). Cholesteric liquid-crystalline DNA–a new type of chemical detector of ionizing radiation. Liq. Cryst..

[16] Kolyvanova M.A., Klimovich M.A., Belousov A.V., Kuzmin V.A., Morozov V.N. (2022). A principal approach to the detection of radiation-induced DNA damage by circular dichroism spectroscopy and its dosimetric application. Photonics.

[17] Yevdokimov Y., Skuridin S., Salyanov V., Semenov S., Kats E. (2019). Liquid-crystalline dispersions of double-stranded DNA. Crystals.

[18] Ramos J.E.B., de Vries R., Neto J.R. (2005). DNA psi-condensation and reentrant decondensation: Effect of the PEG degree of polymerization. J. Phys. Chem. B.

[19] Oh Y.S., Park J.H., Han S.W., Kim S.K., Lee Y.A. (2018). Retained binding mode of various DNA-binding molecules under molecular crowding condition. J. Biomol. Struct. Dyn..

[20] Goldar A., Thomson H., Seddon J.M. (2007). Structure of DNA cholesteric spherulitic droplet dispersions. J. Phys. Condens. Matter..

[21] Leonard M., Hong H., Easwar N., Strey H.H. (2001). Soft matter under osmotic stress. Polymer.

[22] Stanley C.B., Hong H., Strey H.H. (2005). DNA cholesteric pitch as a function of density and ionic strength. Biophys. J..

[23] Yevdokimov Y.M., Skuridin S.G., Salyanov V.I., Volkov V.V., Dadinova L.A., Kompanets O.N., Kats E.I. (2015). About the spatial organization of double-stranded DNA molecules in the cholesteric liquid-crystalline phase and dispersion particles of this phase. Biophysics.

[24] Yevdokimov Y.M., Skuridin S.G., Salyanov V.I., Kats E.I. (2016). Temperature-induced changes of the packing of double-stranded linear DNA molecules in particles of liquid-crystalline dispersions. Biophysics.

[25] Sakurai S., Jo K., Kinoshita H., Esumi M., Tanaka M. (2020). Guanine damage by singlet oxygen from SYBR Green I in liquid crystalline DNA. Org. Biomol. Chem..

[26] Belyakov V.A., Orlov V.P., Semenov S.V., Skuridin S.G., Yevdokimov Y.M. (1996). Comparison of calculated and observed CD spectra of liquid crystalline dispersions formed from double-stranded DNA and from DNA complexes with coloured compounds. Liq. Cryst..

[27] Skuridin S.G., Dubinskaya V.A., Rudoy V.M., Dement’eva O.V., Zakhidov S.T., Marshak T.L., Kuzmin V.A., Popenko V.I., Yevdokimov Y.M. (2010). Effect of gold nanoparticles on DNA package in model systems. Dokl. Biochem. Biophys..

[28] Yevdokimov Y.M., Skuridin S.G., Salyanov V.I., Damaschun G., Damaschun H., Misselwitz R., Kleinwächter V. (1990). Effect of platinum(II) chemotherapeutic agents on properties of DNA liquid crystals. Biophys. Chem..

[29] Yevdokimov Y.M., Skuridin S.G., Salyanov V.I., Kats E.I. (2018). Anomalous behavior of the DNA liquid-crystalline dispersion particles and their phases. Chem. Phys. Lett..

[30] Dragan A.I., Pavlovic R., McGivney J.B., Casas-Finet J.R., Bishop E.S., Strouse R.J., Schenerman M.A., Geddes C.D. (2012). SYBR Green I: Fluorescence properties and interaction with DNA. J. Fluoresc..

[31] Bazhulina N.P., Nikitin A.M., Rodin S.A., Surovaya A.N., Kravatsky Y.V., Pismensky V.F., Archipova V.S., Martin R., Gursky G.V. (2009). Binding of Hoechst 33258 and its derivatives to DNA. J. Biomol. Struct. Dyn..

[32] Morozov V.N., Klimovich M.A., Kolyvanova M.A., Dement’eva O.V., Rudoy V.M., Kuzmin V.A. (2021). Interaction of gold nanoparticles with cyanine dyes in cholesteric DNA submicroparticles. High Energy Chem..

[33] Livolant F. (1991). Ordered phases of DNA in vivo and in vitro. Physica A.

[34] Livolant F., Leforestier A. (1996). Condensed phases of DNA: Structures and phase transitions. Prog. Polym. Sci..

[35] Durand D., Doucet J., Livolant F. (1992). A study of the structure of highly concentrated phases of DNA by X-ray diffraction. J. Phys. II Fr..

[36] Budker V.G., Slattum P.M., Monahan S.D., Wolff J.A. (2002). Entrapment and condensation of DNA in neutral reverse micelles. Biophys. J..

[37] Morozov V.N., Kuzmin V.A. (2019). Fluorescence self-quenching of Hoechst 33258 and SYBR Green I dyes in a DNA cholesteric liquid-crystalline matrix. High Energy Chem..

[38] Spink C.H., Chaires J.B. (1999). Effects of hydration, ion release, and excluded volume on the melting of triplex and duplex DNA. Biochemistry.

[39] Petraccone L., Pagano B., Giancola C. (2012). Studying the effect of crowding and dehydration on DNA G-quadruplexes. Methods.

[40] Miller M.C., Buscaglia R., Chaires J.B., Lane A.N., Trent J.O. (2010). Hydration is a major determinant of the G-quadruplex stability and conformation of the human telomere 3′ sequence of d(AG3(TTAG3)3). J. Am. Chem. Soc..

[41] Nakano S.I., Karimata H., Ohmichi T., Kawakami J., Sugimoto N. (2004). The effect of molecular crowding with nucleotide length and cosolute structure on DNA duplex stability. J. Am. Chem. Soc..

[42] Miyoshi D., Karimata H., Sugimoto N. (2006). Hydration regulates thermodynamics of G-quadruplex formation under molecular crowding conditions. J. Am. Chem. Soc..

[43] Saenger W. (1987). Structure and dynamics of water surrounding biomolecules. Annu. Rev. Biophys. Biophys. Chem..

[44] Preisler R.S., Chen H.H., Colombo M.F., Choe Y., Short B.J., Rau D.C. (1995). The B form to Z form transition of poly(dG-m5dC) is sensitive to neutral solutes through an osmotic stress. Biochemistry.

[45] Barciszewski J., Jurczak J., Porowski S., Specht T., Erdmann V.A. (1999). The role of water structure in conformational changes of nucleic acids in ambient and high-pressure conditions. Eur. J. Biochem..

[46] Ferreira J.M., Sheardy R.D. (2018). Linking temperature, cation concentration and water activity for the B to Z conformational transition in DNA. Molecules.

[47] Nakano S.I., Wu L., Oka H., Karimata H.T., Kirihata T., Sato Y., Fujii S., Sakai H., Kuwahara M., Sawai H. (2008). Conformation and the sodium ion condensation on DNA and RNA structures in the presence of a neutral cosolute as a mimic of the intracellular media. Mol. Biosyst..

[48] Phromsiri P., Gerling R.R., Blose J.M. (2017). The effects of a neutral cosolute on the B to Z transition for DNA duplexes incorporating both CG and CA steps. Nucleosides Nucleotides Nucleic Acids.

[49] Yevdokimov Y.M., Skuridin S.G., Akimenko N.M. (1984). Liquid-crystal microphases of low-molecular weight double stranded nucleic acids and synthetic polynucleotides. Polym. Sci. (USSR).

[50] Bhanjadeo M.M., Baral B., Subudhi U. (2019). Sequence-specific B-to-Z transition in self-assembled DNA: A biophysical and thermodynamic study. Int. J. Biol. Macromol..

[51] Cheng S.M., Mohr S.C. (1975). Condensed states of nucleic acids. II. Effects of molecular size, base composition, and presence of intercalating agents on the psi transition of DNA. Biopolymers.

[52] Widom J., Baldwin R.L. (1983). Inhibition of cation-induced DNA condensation by intercalating dyes. Biopolymers.

[53] Rocha M.S., Cavalcante A.G., Silva R., Ramos E.B. (2014). On the effects of intercalators in DNA condensation: A force spectroscopy and gel electrophoresis study. J. Phys. Chem. B.

[54] Chaires J.B. (1983). Daunomycin inhibits the B → Z transition in poly d(G-C). Nucleic Acids Res..

[55] Mirau P.A., Kearns D.R. (1983). The effect of intercalating drugs on the kinetics of the B to Z transition of poly(dG-dC). Nucleic Acids Res..

[56] Hur J.H., Lee A.R., Yoo W., Lee J.H., Kim K.K. (2019). Identification of a new Z-DNA inducer using SYBR green 1 as a DNA conformation sensor. FEBS Lett..

[57] Yoshinaga N., Akitaya T., Yoshikawa K. (2001). Intercalating fluorescence dye YOYO-1 prevents the folding transition in giant duplex DNA. Biochem. Biophys. Res. Commun..

[58] Kiser J.R., Monk R.W., Smalls R.L., Petty J.T. (2005). Hydration changes in the association of Hoechst 33258 with DNA. Biochemistry.

[59] Anuradha, Alam M.S., Chaudhury N.K. (2010). Osmolyte changes the binding affinity and mode of interaction of minor groove binder Hoechst 33258 with calf thymus DNA. Chem. Pharm. Bull..

[60] Tverdislov V.A., Malyshko E.V. (2019). On regularities in the spontaneous formation of structural hierarchies in chiral systems of nonliving and living matter. Phys. Usp..

[61] Kompanets O.N. (2004). Portable optical biosensors for the determination of biologically active and toxic compounds. Phys. Usp..

[62] Ouameur A.A., Tajmir-Riahi H.A. (2004). Structural analysis of DNA interactions with biogenic polyamines and cobalt(III)hexamine studied by Fourier transform infrared and capillary electrophoresis. J. Biol. Chem..

[63] Zipper H., Brunner H., Bernhagen J., Vitzthum F. (2004). Investigations on DNA intercalation and surface binding by SYBR Green I, its structure determination and methodological implications. Nucleic Acids Res..

[64] Singhal G., Rajeswari M.R. (2010). Molecular aspects of the interaction of Hoechst-33258 with GC-rich promoter region of c-met. DNA Cell Biol..

